# Effectiveness of a 6-Month Multicomponent Training on Physical Frailty in Older Adults With Cognitive Impairment

**DOI:** 10.1093/geroni/igaa057.565

**Published:** 2020-12-16

**Authors:** Oscar Ribeiro, Flavia Borges-Machado, Duarte Barros, Joana Carvalho

**Affiliations:** 1 Department of Education and Psychology, University of Aveiro, Aveiro, Portugal; 2 Faculty of Sports, University of Porto, Porto, Portugal

## Abstract

Multicomponent training (MT) that combines aerobic, strength, postural, and balance exercises seems to be safe and effective to prevent and even reverse early stages of frailty, as well as counteract the physical consequences of cognitive impairment. This study analyzes the effectiveness of MT on physical frailty in older adults diagnosed with cognitive impairment. Forty-nine subjects (34 women) diagnosed with mild cognitive impairment (MCI) or dementia were allocated into exercise group (EG; 75.80±6.60 years; age range: 61-90) or a control group (CG; 82.56±5.45 years; age range: 73-90). EG was submitted to a 6-month MT intervention (2x/week, 50min). CG had a monthly recreational session. Sample mean of MMSE at baseline was 21.02 (±4.98). Participants were categorized as frail, pre-frail and robust according to a validated physical performance battery - Short Performance Physical Battery (SPPB). Overall, in baseline there were 12.5% frail and 37.5% pre-frail individuals in the EG group; in the CG there were 36% and 32%, respectively. Data from SPPB total score revealed that EG group increased the performance over time [t(23)=3,94; Δ=1.21 (±1.50); p<0.001], as opposite to CG individuals [t(24)=-2.26; Δ=-0.92 (±2.04); p=0.034]; also, there were 8.3% frail and 29.2% pre-frail in EG, and 40% and 32% in CG after intervention. By improving lower extremity functionality of patients diagnosed with cognitive impairment, MT intervention may be an important non-pharmacological strategy to improve physical frailty status and modify subjects’ condition from frail to pre-frail or even robust. Supported by IPDJ & FCT: CIAFEL (FCT/UIDB/00617/2020), “Body and Brain” (POCI-01-0145-FEDER-031808), PhD Grant (SFRH/BD/136635/2018).

